# *Gelidocalamus xunwuensis* (Poaceae, Bambusoideae), a new species from southeastern Jiangxi, China

**DOI:** 10.3897/phytokeys.85.13804

**Published:** 2017-08-31

**Authors:** Wen-Gen Zhang, Xue-Nan Ji, Yu-Guang Liu, Wei-Jian Li, Guang-Yao Yang

**Affiliations:** 1 Jiangxi Provincial Key Laboratory for Bamboo Germplasm Resources and Utilization, Forestry College, Jiangxi Agricultural University, Nanchang 330045, P. R. China; 2 Collaborative Innovation Center of Jiangxi Typical Trees Cultivation and Utilization, Nanchang 330045, P. R. China

**Keywords:** Arundinarieae, Bambusoideae, bamboo, leaf epidermis, SEM, taxonomy

## Abstract

*Gelidocalamus xunwuensis* W.G.Zhang & G.Y.Yang, a new species collected from Xunwu County of Jiangxi Province in China, is described and illustrated. The new species is similar to *G. stellatus* in the habit, but differs by internodes sparsely hairy with granuliferous warts, culm sheath stiffly hairy, culm sheath blade broadly lanceolate to narrowly triangular, each node with a ring of appressed trichomes below, foliage leaves broadly lanceolate to narrowly oblong, and new shoots occurring in late October.

## Introduction

The genus *Gelidocalamus* Wen (1982: 21) includes ca. 9-13 species in the tribe Arundinarieae (Poaceae: Bambusoideae) and is endemic to China. This genus is characterized by leptomorph rhizomes, several branches per node, leaves usually solitary on each ultimate branch, semelauctant inflorescence, three stamens, and with new shoots occurring in the autumn to winter seasons (Wen 1982, Geng and Wang 1996, Li et al. 2006, Yi et al. 2008, Soreng et al. 2015, Liu et al. 2017).

Most species of *Gelidocalamus* are restricted to southern China in Hunan, Jiangxi, Zhejiang, Fujian, Taiwan, Guangdong, Guangxi, Yunnan, and Guizhou Provinces, and distributed at elevations of 200–1200 m, along ravines and under evergreen broad-leaved forests (Li et al. 2016). However, within the past 30 years, most newly discovered species, e.g. *Gelidocalamus annulatus* T. H. Wen, *G. longiinternodus* T. H. Wen & S. C. Chen, *G. multifolius* B. M. Yang and *G. dongdingensis* C. F. Huang & C. D. Dai, are known only from their type locations suggesting that the diversity and distribution of *Gelidocalamus* species is in need of further study.

During a botanical expedition in central and southern China in 2014, a distinctive “*Gelidocalamus*-like” collection with many branches per node and leaf solitary on each ultimate branch was found from Xunwu County. Xunwu County (24°30'40"–25°12'10"N, 115°21'22"–115°54'25"E) is a hot and humid region in the southeastern corner of Jiangxi Province, located at the junction of Wuyi Mountain and Jiulian Mountain, and has a subtropical climate with abundant monsoon rainfall. It is also a minor centre of plant endemism in China and especially exhibits high richness in palaeo-endemic species (Jordi et al. 2011). Twenty-six endemic species and 11 new species have been discovered in this region in recent years (Ji 2007, Ji et al. 2010).

To investigate this collection, we made a complete morphological characterization, including description, illustrations, taxonomic comments, and scanning electron microscope (SEM) images of the abaxial leaf epidermis. This collection has the typical characteristics of *Gelidocalamus* with leptomorph rhizomes, several branches per node and leaves usually solitary on each ultimate branch. It can be readily distinguished from other *Gelidocalamus* species by its internodes being sparsely hairy with granuliferous warts, culm sheath stiffly hairy, and foliage leaves broadly lanceolate to narrowly oblong. By all the evidence obtained, we believe that this collection is a new species and herein described and illustrated.

## Materials and methods

From Jul. 2014 to Nov. 2016, mature leaves were collected from individuals of the type localities (the Xunwu population – the Guizhumao of Xunwu in Jiangxi, *Gelidocalamus stellatus* T. H. Wen – the Xiazhuang of Jinggang Mountain in Jiangxi, *G. tessellatus* T. H. Wen & C. C. Chang – the Maolan of Libo in Guizhou, *G. dongdingensis* C. F. Huang & C. D. Dai – the Dongding Mountain of Longyan in Fujian, respectively) and immediately fixed in FAA solution. Leaves were cleaned by ultrasonic wave with ultrapure water, dried at room temperature, and mounted on stubs. After gold sputtering, the samples were photographed using the scanning electron microscope Hitachi S-4800. Terminology for the epidermis appendages follows Ellis (1979), Wu et al. (2014), and Zhang et al. (2014). Voucher specimens were deposited in the herbarium of the College of Forestry, Jiangxi Agricultural University, China (JXAU). Morphological traits, including habit and new shoot, culm and culm sheath, branch and leaf, were described with both fresh and exsiccated specimens.

## Results

**Figure 1.**
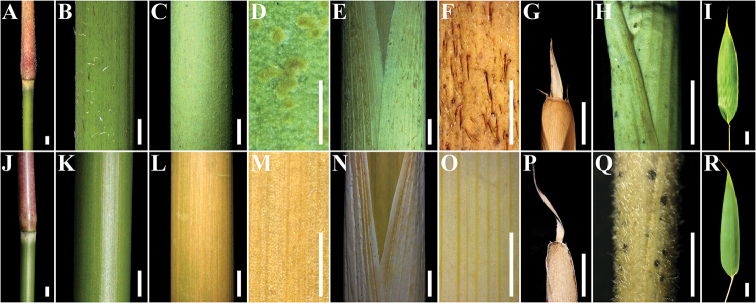
Comparison of morphological characters between plants from Xunwu (**A–I**) and *Gelidocalamus stellatus* (**J–R**). **A–D, J–M** culm and node **E–G, N–P** culm leaf sheath **H, Q** branch sheath **I, R** foliage leaf. Scale bars: 3 mm (**A–C, E, J–L, N**), 1 mm (**D, F–H, M, O–Q**), 2 cm (**I, R**).

The Xunwu population plants are most similar to *G. stellatus* in the habit and branching pattern. However, they differ from the latter by the following characters: culms sparsely hairy (vs. glabrous) with granuliferous warts (vs. smooth), culm leaf sheath densely hispidulous (vs. hairless), each node with a ring of fulvous appressed trichomes below (vs. white appressed trichomes), foliage leaves oblong (vs. lanceolate) and new shoot in late Oct. (vs. early Sep.) (Table 1 and Fig. 1).

**Figure 2.**
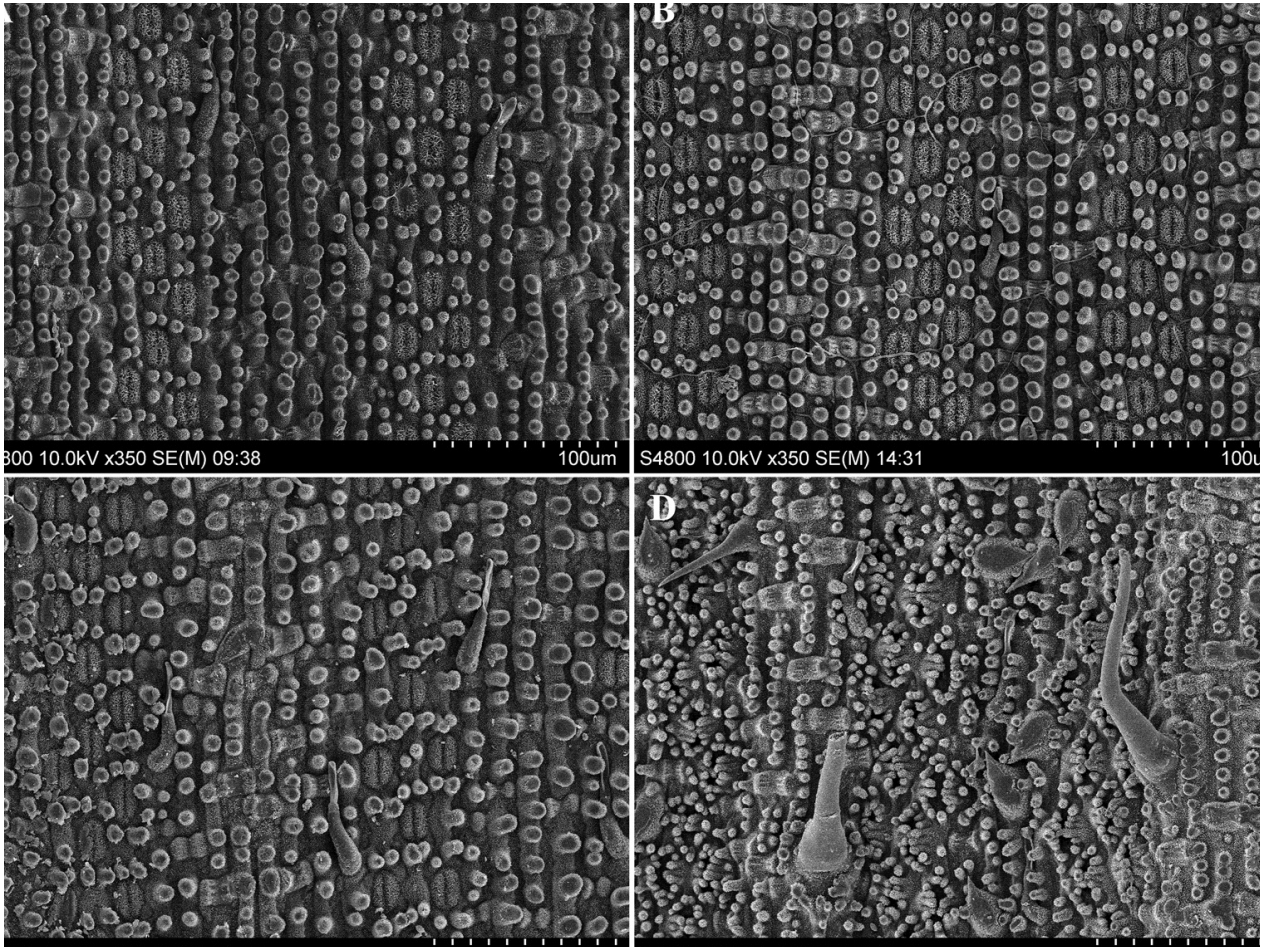
SEM images of the abaxial leaf epidermis. **A** Xunwu plants (Xunwu, Jiangxi, China) **B** *Gelidocalamus stellatus* (Jinggang Mountain, Jiangxi, China). **C** *Gelidocalamus tessellatus* (Libo, Guizhou, China) **D** *Gelidocalamus dongdingensis* (Longyan, Fujian, China).

Leaf epidermis characters of the Xunwu population plants are almost identical to that of *Gelidocalamus stellatus* Wen (1982: 22) and *G. tessellatus* T. H. Wen & C. C. Chang (1982: 24) (Fig. 2A–C). Each stomatal apparatus (usually 3 rows between the veins) is surrounded by 8–10 short papillae, but covered with dense wax. The saddle-shaped silica bodies on the veins can be clearly identified, but the long cells and short cells cannot be distinguished. Microhairs are gracile, composed of two cells with the apical cell withered, and mostly occur on the intercostal regions of the abaxial leaf epidermis.

On the contrary, *Gelidocalamus dongdingensis*, a species collected from the adjacent area of Xunwu County, Longyan of Fujian (about 200 km), has obviously different characters of leaf epidermis (Fig. 2D). Each stomatal apparatus (usually 8–10 rows between the veins) is nearly invisible, overarched by 14–20 long papillae. The saddle-shaped silica bodies on the veins can be also clearly identified. However, there are three types of trichomes (i.e. macrohairs, microhairs and prickles) on the abaxial leaf epidermis. Except microhairs, macrohairs can be visualized with the naked eye and can be used to discriminate from the Xunwu population plants, while prickles are short and relatively stiff with sharp apices, located at the intercostal regions.

**Table 1.** Comparison of morphological traits of the Xunwu population plants with those of *Gelidocalamus stellatus*.

Characters	Xunwu Plants	*Gelidocalamus stellatus*
Culm	internodes rough with granuliferous warts, strigose; each node with a ring of fulvous appressed trichomes below	internodes glabrous; each node with a ring of white appressed trichomes below
Branch	branching intravaginal from 4^th^ node up, usually 4–9 on mid-culm	branching intravaginal from 7^th^ node up, usually 6–12 on mid-culm
Culm leaf	culm leaf sheath carmine, densely pubescent with stiffly dark-brown hairs; oral setae 3–5–paired, ca. 2–5 mm; blade deciduous, narrowly triangular or linear-lanceolate, ca. 1/3–1/2 as wide as sheath apex	culm sheath purple-red, glabrous; oral setae absent or weak; blade deciduous, linear or linear-lanceolate, ca. 1/5–1/4 as wide as sheath apex
Foliage leaf	leaf blade broadly lanceolate to narrowly oblong, usually 15–20×2.3–3.0 cm, pubescent near base, basally slightly revolute and symmetrical	leaf blade lanceolate to narrowly lanceolate, usually 14–19×2.1–2.7 cm, basally cuneate and asymmetrical
New shoot	late October	early September

## Discussion

Recently, phylogenetic studies have indicated that *Gelidocalamus* is polyphyletic (Zeng et al. 2010, Zhang et al. 2012). Four species (*G. tessellatus*, *G. rutilans* T. H. Wen and two unnamed species) were examined by Zeng et al. (2010) and were found to be widely divergent. However, few species have been examined molecularly and more extensive sampling within *Gelidocalamus* is necessary. Similarly, studies of leaf micromorphology show that there are various patterns of the papillae surrounding the stomata among species of *Gelidocalamus* (Wu et al. 2014, Zhang et al. 2014, Liu et al. 2017).

In contrast, previous studies have also shown that leaf epidermis characters are almost identical among the main taxa of *Gelidocalamus*, e.g. *G. stellatus*, *G. tessellatus*, *G. annulatus*, *G. multifolius*, *G. latifolius* T. H. Wen (1985: 53) (Wu et al. 2014, Zhang et al. 2014, Long et al. 2015). The stomatal apparatuses are embossed outwards and usually surrounded by ca. 6–10 short papillae, obviously distinguished from those of other taxa, such as *G. subsolidus* W. T. Lin & Z. J. Feng (1990 : 18), *G. solidus* C. D. Chu & C. S. Chao (1984:75), *G. rutilans*, *G. monophyllus* (Yi & B. M. Yang) B. M. Yang (1989: 338), *G. kunishii* (Hayata) P. C. Keng & T. H. Wen (1983: 20) and *G. dongdingensis* (Fig. 2D) (Wu et al. 2014, Zhang et al. 2014, Liu et al. 2017), indicating that the genus *Gelidocalamus* may be good after eliminating several taxa.

In the present study, the Xunwu population has the typical characteristics of *Gelidocalamus*, and can be readily distinguished from other *Gelidocalamus* species as observed above. Based on the unique morphological characters, and possibly the disjunct distribution of the new species, we believe that the Xunwu population represents a new species, and is herein described and illustrated.

## Taxonomic treatment

### Gelidocalamus xunwuensis

Taxon classificationPlantaePoalesPoaceae

W.G.Zhang & G.Y.Yang sp. nov.

urn:lsid:ipni.org:names:77165358-1

Figs 3 , 4

#### Diagnosis.

Similar to *G. stellatus* Wen (1982: 22) in the habit and branch, but differs by culms sparsely hairy (early period) with granuliferous warts (adult or later period), each node with a ring of fulvous appressed trichomes below, culm leaf sheath densely hispidulous with a blade broadly lanceolate and 3–5–paired oral setae, branch sheath glabrous, foliage leaves broadly lanceolate to narrowly oblong, and new shoots late October.

#### Type.

**Figure 3.**
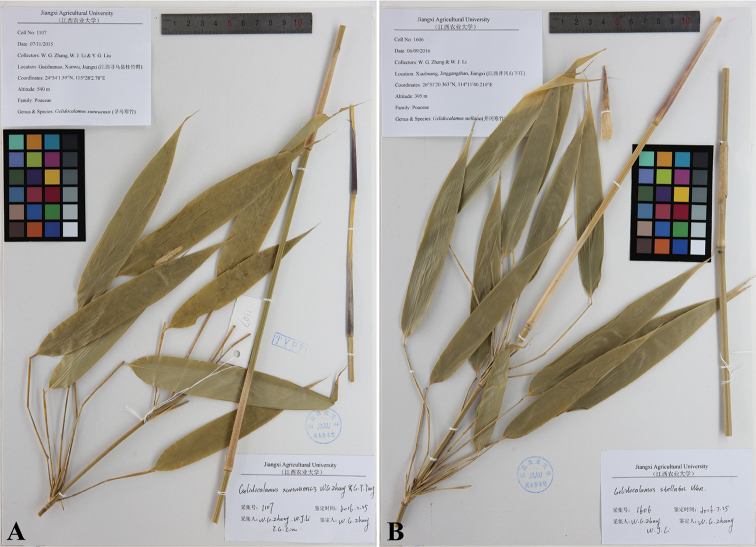
*Gelidocalamus xunwuensis* and *Gelidocalamus stellatus*. **A** *G. xunwuensis*, China, Jiangxi, Xunwu County, Guizhumao, *W.G. Zhang et al. 1107* (holotype, JXAU!), with culm leaf sheath stiffly hairy and foliage leaf broadly lanceolate to narrowly oblong **B** *G. stellatus*, China, Jiangxi, Jinggangshan, Xiazhuang, *W.G. Zhang & W.J. Li 1606* (JXAU!), with culm leaf sheath glabrous and foliage leaf lanceolate to narrowly lanceolate.

CHINA. Jiangxi Province: Xunwu County, 24°54'1.59"N, 115°28'2.78"E, elev. ca. 540 m, 7 Nov. 2015, *W.G. Zhang et al. 1107* (holotype: JXAU!) (Fig. 3A).

#### Description.

Rhizomes leptomorph. Culms up to 2.5 m tall, ca. 2.0–5.6 mm in diam., erect, apically slightly nodding; internodes rough strigose with granuliferous warts, 12–20 cm long, wall 0.5-1.5 mm thick; each node with a ring of fulvous appressed hairs below and above sheath scar; nodal line upheaving markedly above 3–5 unequal buds, supranodal ridge present and prominent. Branching intravaginal from 4^th^ node up, ca. 4–9 branches each node; branches equal or subequal, ca. 8–32 cm long, 1–2 mm in diam. Culm leaf sheaths tardily deciduous, 7–10 cm, abaxially carmine and densely hispidulous when young, then grey-white when old, ribbed-striate, pubescent with stiffly dark-brown hairs, apex slightly oblique and truncate; auricles absent or weak; oral setae curved, ca. 2–5 mm, 3–5–paired; ligule truncate, ca. 1 mm, scabrous, very shortly finely fimbriate; blade deciduous, narrowly triangular or linear-lanceolate, 10–15×0.9–1.2 mm, erect or recurved, apex acuminate, base blunt or truncate, ca. 1/3–1/2 as wide as sheath apex, margins scabrous. Ultimate branches usually with 1 foliage leaf; branch sheath glabrous; ligule truncate, ca. 1 mm, scabrous; auricles absent or weak; oral setae erect or curved; leaf blade narrowly oblong, ca. 15–20×2.3–3.0 cm, abaxially pubescent near base, apically acuminate, basally cuneate and symmetrical, margins serrulate and slightly revolute near base.

#### Etymology.

The species epithet *xunwuensis* refers to the locality of the type specimen: Xunwu County, Jiangxi, China.

#### Phenology.

New shoots late October; flowering unknown.

#### Distribution and habitat.

*Gelidocalamus xunwuensis* occurs under evergreen broad-leaved forests, along ravine, and roadsides at elev. ca. 400–600 m. It grows together with *Castanopsis kawakamii* Hay., *Dicranopteris pedata* (Houtt.) Nakaike, *Gnetum parvifolium* (Warb.) C. Y. Cheng & Chun, *Eurya chinensis* R. Br., *Semiliquidambar cathayensis* H. T. Chang, and *Ormosia semicastrata* Hance. *Gelidocalamus xunwuensis* is currently known from only one small populations (less than 100 culms) in the southern China.

#### Leaf micromorphology.

Stomatal apparatuses, ca. 22 (20–24) × 12 (10–14) µm, are embossed outwards and covered by platelet-like wax. Short papillae occur on the abaxial leaf epidermis and appear randomly around the stomata (ca. 6–10 short papillae). Microhairs are composed of two cells with the apical cell withered, mostly distributed on the intercostal regions of abaxial epidermis. Silica bodies are saddle-shaped on the veins and can be clearly identified (Fig. 2A).

#### Conservation status.

As a running bamboo, the new species is difficult to count each individual. Using the World Conservation Union Red List Categories and Criteria (IUCN 2012), *G. xunwuensis* should be treated as a data deficient species with less than 100 culms in the type locality.

**Figure 4.**
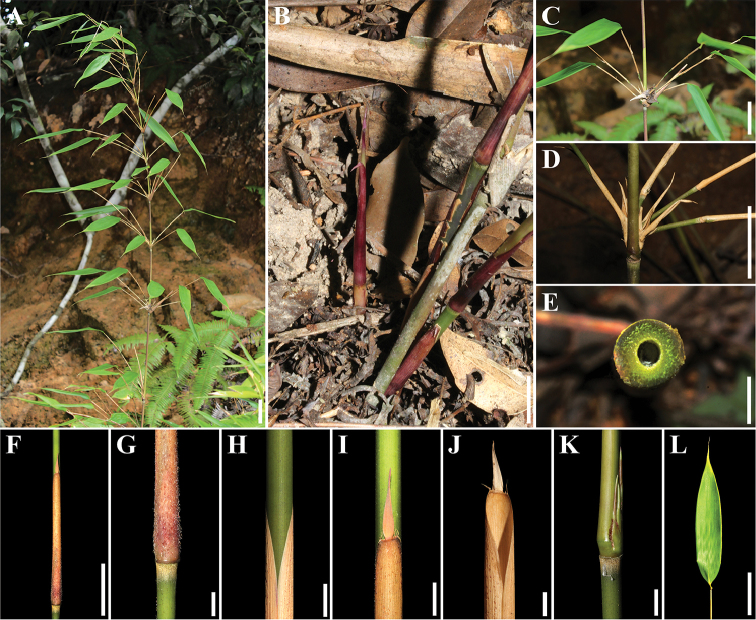
*Gelidocalamus xunwuensis*. **A** habitat plants **B** new shoot **C–L** detailed characters, show branch and branch sheath (**C–D**), transection of culm and pith-cavity (**E**), culm and its leaf sheath (**F–J**), buds (**K**) and foliage leaf (**L**). Scale bar: 5 cm **(A–D, F, L**), 5 mm (**E, G–K**).

## Supplementary Material

XML Treatment for Gelidocalamus xunwuensis
